# The cholesterol-lowering effect of statins is modified by *LILRB5* intolerance genotype: Results from a recruit-by-genotype clinical trial

**DOI:** 10.3389/fphar.2023.1090010

**Published:** 2023-03-14

**Authors:** Aleksi Tornio, Margherita Bigossi, Moneeza K. Siddiqui, Gwen Kennedy, Ala’a Melhem, Mehul K. Chourasia, Cyrielle Maroteau, Roberto Pola, Daniel I. Chasman, Alexander S. F. Doney, Colin N. A. Palmer

**Affiliations:** ^1^ Integrative Physiology and Pharmacology, Institute of Biomedicine, University of Turku, Turku, Finland; ^2^ Unit of Clinical Pharmacology, Turku University Hospital, Turku, Finland; ^3^ Pat Macpherson Centre for Pharmacogenomics and Pharmacogenetics, Division of Population Health & Genomics, School of Medicine, University of Dundee, Dundee, United Kingdom; ^4^ Section of Internal Medicine and Thromboembolic Diseases, Department of Medicine, Fondazione Policlinico Universitario A. Gemelli IRCCS, Rome, Italy; ^5^ Division of Preventive Medicine, Brigham and Women’s Hospital, Harvard Medical School, Boston, MA, United States

**Keywords:** RCT-randomized controlled trial, ADR (adverse drug reaction), statin (HMG-CoA reductase inhibitor), treg-regulatory T cell, non-HDL cholesterol

## Abstract

**Background/Aims:** Statin intolerance leads to poor adherence to statin therapy, resulting in a failure to achieve desired cholesterol reduction and adverse outcomes. The *LILRB5* Asp247Gly genotype has been identified as being associated with statin intolerance and statin-induced myalgia. We conducted a randomized clinical trial to examine its role in immune response through T regulatory cell aggregation and in achieving cholesterol reduction targets.

**Methods:** A double-blind, cross-over, recruit-by-genotype trial was undertaken. A total of 18 participants who had either the Asp247Asp (T/T) genotype or the Gly247Gly (C/C) genotype were recruited to the study. Participants were randomised to receive placebo or atorvastatin 80 mg daily for 28 days. Following a washout period of 3 weeks, they were then switched to the opposite treatment. Biochemical and immunological measurements as well as interviews were performed prior to and after both treatment periods. Within genotype group comparisons were performed using repeated measures Wilcoxon tests. Two-way repeated measures ANOVA with genotype and treatment as factors were used to compare changes in biochemical parameters between groups during placebo and atorvastatin periods.

**Results:** Individuals with the Asp247Asp genotype had a greater increase in creatine kinase (CK) compared to those with Gly247Gly genotype in response to atorvastatin (*p* = 0.03). Those with Gly247Gly genotype had a mean non-HDL cholesterol reduction of 2.44 (95% CI:1.59 – 3.29) mmol/L while in Asp247Asp genotype group the mean reduction was 1.28 (95%CI: 0.48 – 2.07) mmol/L. The interaction between the genotype and atorvastatin treatment for total cholesterol (p = 0.007) and non-HDL cholesterol response was significant (p = 0.025). Immunological assessment showed no significant changes in aggregation of T regulatory cells by genotype.

**Conclusion:** The Asp247Gly variant in *LILRB5*, previously associated with statin intolerance, was associated with differential increases in creatine kinase and total cholesterol and non-HDL cholesterol-lowering response to atorvastatin. Taken together, these results suggest that this variant could have utility in precision cardiovascular therapy.

## Introduction

Statins, 3-hydroxy-3-methylglutaryl-coenzyme-A reductase inhibitors, are the most widely used lipid-modifying agents worldwide for prevention of cardiovascular diseases. Even though statins are generally well-tolerated, they are associated with muscle symptoms, ranging from common muscle pain (myalgia) to rarely muscle damage (myopathy) (Alfirevic et al., 2014), both typically reversible. Even mild muscle symptoms can negatively impact adherence to statin-therapy, limiting the benefit in real-world settings (Guyton et al., 2014; Rannanheimo et al., 2015). Poor adherence to statins limits efficacy and results in higher risk of major adverse cardiovascular events (MACE) through limited reduction in cholesterol.

Leukocyte immunoglobin-like receptor subfamily-B 5 (*LILRB5*) is a member of the leukocyte immunoglobin-like receptor (LILR) family, a group of receptors expressed on the surface of immune cells and exerting activating or inhibitory function. rs12975366T>C is a common (mean allele frequency 0.40) missense variant in *LILRB5* resulting in an aspartate to glycine amino acid substitution (p.Asp247Gly). Asp247Gly was reported to be associated with circulating levels of Creatine kinase (CK) and lactate dehydrogenase (LDH), both markers of muscle breakdown. In 2017, the variant was reported to be associated with statin intolerance in a case-control study with increased odds of having raised creatine kinase (CK) and being non-adherent to statin therapy. The effect of the genotype was also found to be dominant. Those with the LILRB5 Asp247Asp (T/T) genotype had 1.34 times the odds of statin intolerance compared to those with the Gly247X (T/C or C/C) genotype (Siddiqui et al., 2017). The study suggested a potential role for the immune system in the development of statin intolerance and myalgia using both observational data and *post hoc* analyses of a clinical trial. The proposed hypothesis was that carriers of the Asp247Asp genotype had reduced expression of Foxp3+ T regulatory cells, which resulted in poorer muscle repair and regeneration compared to non-carriers (Fontenot et al., 2003; Rodriguez-Perea et al., 2015; Kuswanto et al., 2016). This effect was recently replicated in the ODYSSEY outcomes trial of statin-related adverse drug reactions (ADRs) (Murphy et al., 2022). While the observed effect on statin intolerance was replicated in observational, clinically adjudicated, and clinical trial datasets, the studies were retrospective in nature and mechanistic explanation for the association was not directly provided.

Therefore, we sought to examine the association of *LILRB5* genotype with statin tolerance and non-HDL-lowering effect in a pilot prospective, recruit-by-genotype trial in healthy volunteers. Furthermore, we examine if there is a differential aggregation of CD4+/Foxp3+ T cells in response to treatment across genotypes.

## Materials and methods

### Study participants

Potential study participants, who had given their written informed consent to be contacted for research purposes, were identified in the Scottish Health Research register (SHARE) and genotyped for the *LILRB5* Asp247Gly variant (rs12975366) (McKinstry et al., 2017). The main inclusion criteria were age between 40 and 69 years, being non-hypercholesterolemic and statin treatment naïve, having White European ethnicity, and being in generally good health. The main exclusion criteria were inability or unwillingness to consent to the study or comply with the protocol, significant disease, regular drug therapy, recent involvement (<30 days) in a clinical trial with investigational medicinal product, premenopausal females, and being a carrier of the rare variant of the *CKM* polymorphism rs11559024 (Wallace et al., 2016; Siddiqui et al., 2017).

A SHARE administrator first contacted subjects tentatively matching the inclusion and exclusion criteria *via* telephone and contact information of those interested to participate were passed on to the research group. A total of 19 participants were enrolled to the trial after giving a written informed consent. The health of the subjects was confirmed during a screening visit by clinical examination, laboratory tests, and medical history. None of the participants used any continuous medication.

### Study design and ethical approval

The study protocol was approved by the East of Scotland Research Ethics Service (record number 16/ES/0128). The study was a randomized, double-blind (to both genotype and treatment), cross-over study consisting of two phases with a minimum of 3 weeks washout period. In each phase, participants ingested either 80 mg atorvastatin (two over-encapsulated 40 mg atorvastatin tablets (Teva Pharmaceuticals Europe B.V) or matching placebo once daily for 28 days. The study drugs were manufactured, and randomisation performed by Tayside Pharmaceuticals (Ninewells Hospital & Medical School, Dundee, United Kingdom). The use of grapefruit juice and grapefruit containing products was prohibited for the duration of study. The participants were asked to avoid moderate to vigorous physical exercise 72 h prior to study visits. Both phases consisted of two visits to the study site, first on day 0 (baseline) and second on day 29. The visits included blood sampling, muscle symptoms questionnaire, and questions regarding any possible concomitant medications or adverse effects. Unused capsules were collected after each phase and counted. Study design is graphically presented in Supplementary Figure S1. The trial was registered at clinicaltrials.gov (NCT02984293).

### Biochemistry and flow cytometry

In both study phases, fasting serum and EDTA whole blood samples were collected on the visits on days 0 and 29. On both visits, full blood count, CK, cholesterol and triglycerides were assayed at NHS Tayside Blood Sciences routine clinical laboratory at Ninewells Hospital and Medical School, Dundee, United Kingdom. In addition, 10 ml K2-EDTA tubes were drawn for flow cytometry and plasma separation. Flow cytometry was performed from fresh whole blood samples only on days 29.

The proposed hypothesis that the Asp247Gly genotype differentially affects T regulatory cell aggregation in response to insults (including statin-induced muscle damage) was tested. For flow cytometry, we used the same definition for T regulatory cells as Rodríguez-Perea et al., i.e., CD4^+^ and FoxP3+ lymphocytes (Rodriguez-Perea et al., 2015). Anti-Human FoxP3 Staining Kit (cat. No 560131) and all other reagents were purchased from BD Biosciences and flow cytometry was performed per manufacturer’s instructions. In short, K2-EDTA whole blood was first lysed with Lysing solution (cat. No 349202) and Human BD Fc Block™ (cat. No 564220) was added. Samples were then stained for up to 30 min with APC-labeled anti-human CD4 (cat. No 555349). For intracellular staining, the cells were fixed and permeabilized using the Human FoxP3 Buffer Set (cat. No 560098) and stained with Alexa Fluor 488 –labelled anti-human FOXP3 (cat. No 560047). The cells were acquired on LSRFortessa Flow Cytometer (BD). Lymphocyte gate was defined by forward and side scatter parameters and 20,000 CD4 positive lymphocytes were acquired from each sample.

### Replication

Replication of findings was sought from the Tayside Bioresource, a large observational cohort based in Tayside, Scotland (McKinstry et al., 2017; Hebert et al., 2018; Siddiqui et al., 2022). This resources links community prescribing records, electronic medical records, and genetic biobanks in the Tayside region of Scotland. We used frequency of prescription encashment in an average of 9 years of follow-up as a proxy of adherence to therapy.

### Statistical methods

Baseline characteristics of genotype T/T and genotype C/C groups were compared using Fisher’s exact test for binary data and t-test for continuous data. Mean (SD) are reported for continuous variables with normal distribution, median (IQR) for continuous variables with non-parametric distribution, and n (%) for categorical variables.

Within-group comparison were evaluated with repeated measures Wilcoxon test. Two-way repeated measures ANOVA with genotype and treatment as factors was used to compare changes in cholesterol levels between groups during placebo and atorvastatin periods. Holm-Šidák methods were used to correct for multiple comparisons. Data from participants who did not complete both phases of the trial were excluded from all statistical analyses. Patients with missing values in either CK levels, total cholesterol, or HDL cholesterol at any of the prespecified measurement time points were not included in the corresponding statistical analysis. All analyses were conducted using R Core Team 2019 and GraphPad Prism 8 for Mac OS X version 8.2.1.

## Results

The characteristics of the subjects in the two genotype groups are shown in Table 1. Of the 19 subjects enrolled in the trial one withdrew due to personal reasons after screening, and one was lost in follow up after completing phase 1. There were no withdrawals due to adverse events.

**TABLE 1:** Description of trial participants at baseline by LILRB5 Asp247Gly genotype.

Variables	Genotype C/C Gly247Gly (n = 9)	Genotype T/T Asp247Asp (n = 8)	*p*-Value
Sex, female (%)	3 (33.3%)	0 (0%)	N.S.
Age, years [mean (SD)]	53.78 (3.87)	56.88 (5.22)	N.S.
Weight, kg [mean (SD)]	74.6 (11.30)	80.9 (13.9)	N.S.
BMI, kg/m^2^ [mean (SD)]	26.08 (4.61)	27.61 (3.15)	N.S.
Systolic blood pressure, mm Hg [mean (SD)]	128.11 (6.37)	141.9 (14.05)	0.018*
Diastolic blood pressure, mm Hg [mean (SD)]	80.0 (2.92)	84.88 (8.04)	N.S.
CK levels at screening, U/L [median (IQR)]	113.0 (68.0, 168.5)	194.5 (65.75, 292.3)	N.S.
Total cholesterol at baseline, mmol/L [mean (SD)]	5.40 (1.18)	5.09 (0.75)	N.S.
Non-HDL cholesterol at baseline, mmol/L [mean (SD)]	3.96 (0.99)	3.54 (0.96)	N.S.
Smoking status			N.S.
Current smoker	2 (22.2%)	2 (25%)	
Previous smoker	4 (44.4%)	2 (25%)	
Never smoker	3 (33.3%)	4 (50%)	
Alcohol Intake per Day			N.S.
<1 drink per day	5 (55.6%)	3 (37.5%)	
1–5 drinks per day	3 (33.3%)	3 (37.5%)	
>6 drinks per day	1 (11.1%)	2 (25%)	
Physical Exercise on a Weekly Basis	6 (66.7%)	7 (87.5%)	N.S.
Medical history			
Cardiac disease	0	0	
Respiratory disease	0	0	
Gastrointestinal disease	2 (22.2%)	1 (12.5%)	
Musculoskeletal disease	0	2 (25%)	

A total of 17 individuals successfully completed the trial of which 9 belonged to the Gly247Gly (C/C) *LILRB5* genotype and 8 to the Asp247Asp (T/T) genotype. The average age, weight and body mass index (BMI) were comparable across the two genotypes. Individuals with the Asp247Asp genotype had significantly higher systolic blood pressure (when not corrected for multiple testing), however diastolic blood pressures were not different across the two genotype groups. At baseline, CK levels were not significantly different, and neither were total cholesterol or non-HDL cholesterol levels. None of the other features tested were different across the two genotype groups. Physical activity defined as weekly exercise was 67% in the Gly247Gly group and 87% in the Asp247Asp group. Two individuals in the Asp247 group compared to zero in the 247Gly group had a history of musculoskeletal disease. A history of gastrointestinal disease was observed in two individuals with 247Gly genotype and one with 247Asp genotype. Overall, there was no history of cardiac or respiratory disease in the trial population.

### Changes in CK and compliance in response to atorvastatin therapy

**FIGURE 1:**
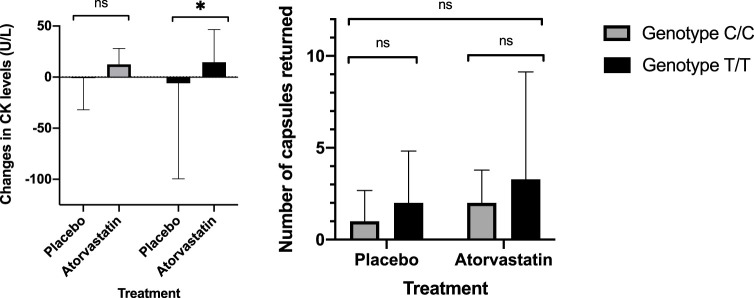
CK changes (left) and number of capsules returned by genotype and treatment group.

We considered three indicators of intolerance or adverse reactions to statin therapy: elevations in CK, poor compliance according to returned capsule count, and reported muscular adverse events. CK levels were increased significantly during treatment compared to placebo in participants with the T/T or Asp247Asp genotype (*p*-value = 0.04, one-tailed *p*-value 0.03), while these were not significantly increased in those with the Gly247Gly genotype (Figure 1A; Supplementary Table S1). When comparing number of capsules returned, more atorvastatin than placebo capsules were returned overall, however, a non-significant trend was observed where those with the T/T genotype returned more atorvastatin capsules compared to placebo capsule in the same genotype group, and in comparison, to the number returned by those with the C/C genotype group (Figure 1B). There were no differences in reported intolerance that differed from baseline complaints of muscular pain. 5 out of 7 participants with the T/T genotype had complaints of non-treatment specific myalgia, while only 3 out of 8 participants with the C/C genotype had the same. However, reports of statin-specific myalgia were made by 2 participants, each belonging to different genotype groups.

### Gly247 carriers have better reduction in total cholesterol and non-HDL cholesterol compared to Asp247

**FIGURE 2:**
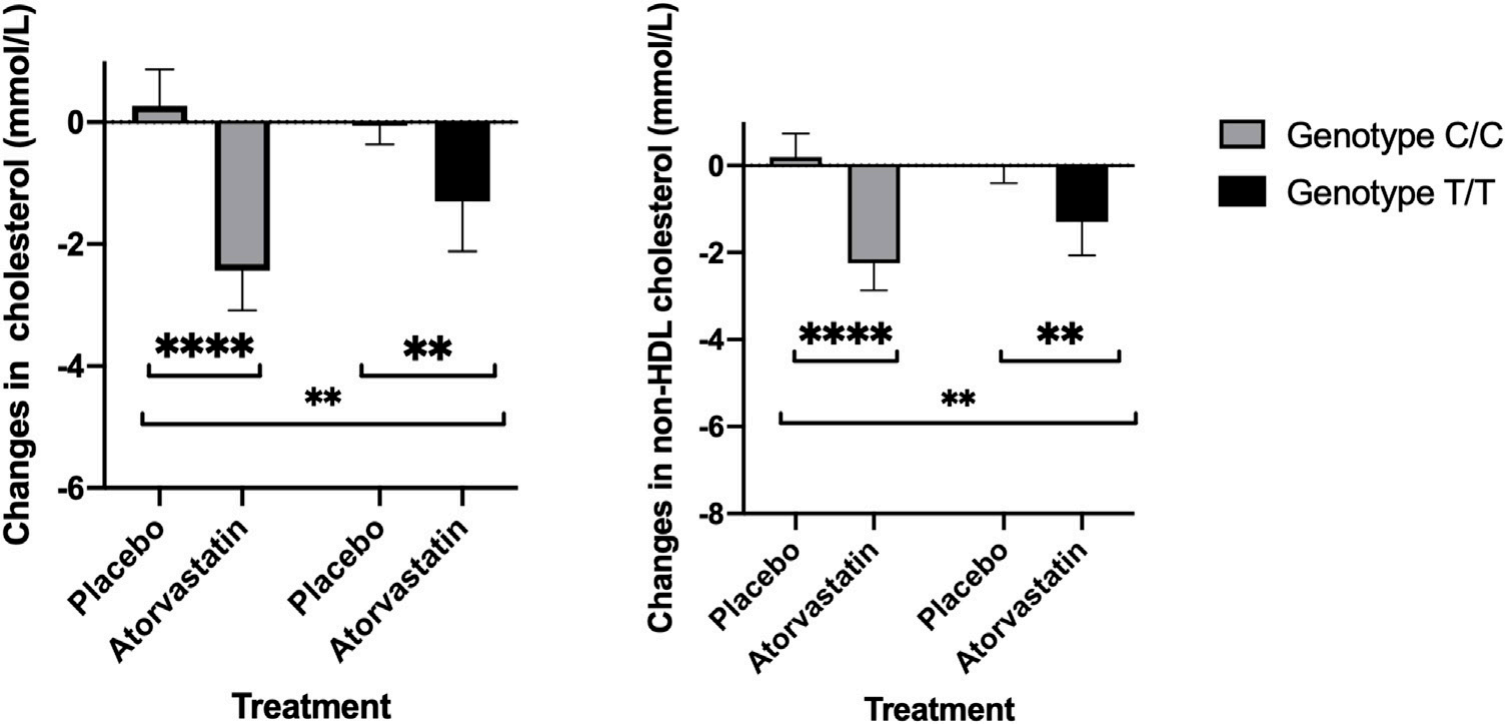
Total cholesterol (left) and non-HDL cholesterol response to placebo and atorvastatin therapy by rs12975366 genotype (Asp247Asp corresponds to T/T and Gly247Gly to C/C).

**FIGURE 3:**
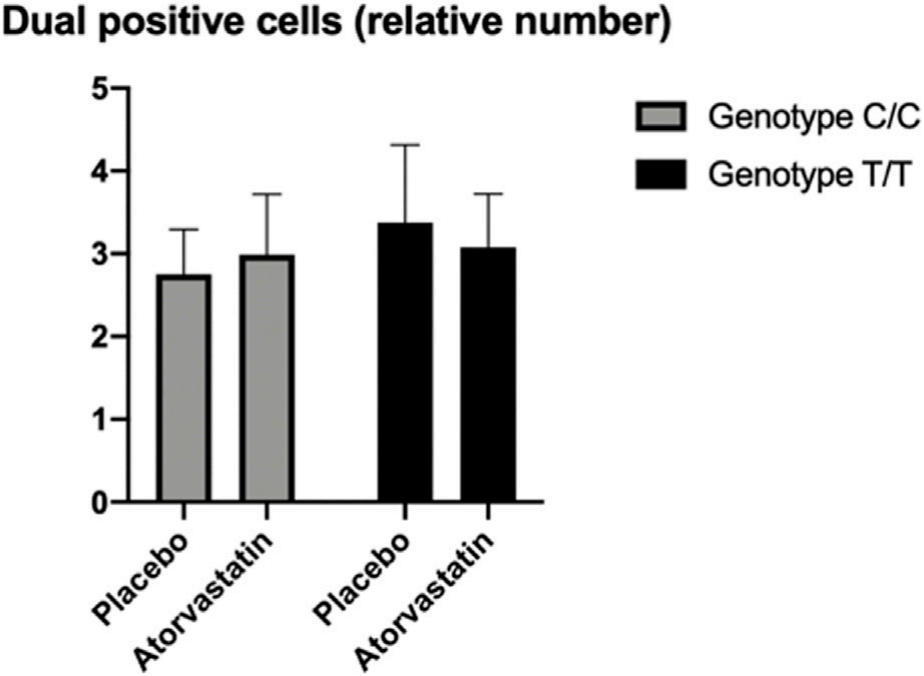
Flow cytometry results for CD4+/Foxp3+ T cells showing non-significant effects of T regulatory cell aggregation differentially by genotype and treatment groups.

Total cholesterol response to atorvastatin therapy differed by genotype. Average reduction in those with Gly247Gly (C/C) genotype was 2.70 (95% CI:1.85 – 3.55) mmol/L while in T/T it was 1.25 (95%CI: 0.45 – 2.04) mmol/L. Statistical interaction between treatment and genotype was also significant (*p* = 0.007) (Figure 3; Supplementary Tables S2, S3). Similarly, non-HDL cholesterol response to treatment differed by genotype (Figure 2). Average reduction in the Gly247 genotype group was 2.44 (95% CI:1.59 – 3.29) mmol/L while in Asp247 group the average response was 1.28 (95%CI: 0.48 – 2.07) mmol/L. The interaction between treatment and genotype was also significant (*p* = 0.025) (Supplementary Tables S4, S5).

### Changes in immune response—T regulatory cells

There was no significant difference in the CD4+/Foxp3+ T cells by genotype following statin therapy. While the results were inconclusive, the average number of cells observed for those with the Gly247 genotype were higher following treatment with statin compared to treatment with placebo. Conversely, the average number of cells observed for the Asp247 genotype group was lower following statin therapy compared to placebo (Figure 3).

### *LILRB5* Asp247Gly is associated with compliance to statin therapy in observational studies

To replicate the observed effects in large observational data, we used the Tayside bioresource where over 80% of statin prescriptions are for simvastatin or atorvastatin. In a cohort of 8591 statin users, Gly247X (T/C or C/C) carriers were more likely than non-carriers to be adherent to their statin therapy (beta: 0.023, *p* = 0.04). This model was adjusted for adherence to statin therapy, changes in dosing regimens, duration of therapy, type 2 diabetes status and a history of cardiovascular disease.

## Discussion

We present the results of a recruit-by-genotype trial to prospectively characterise the effect of *LILRB5* Gly247Asp genotype in healthy volunteers in terms of lipid response, muscle symptoms and T regulatory cell response to high dose atorvastatin therapy. The main finding of the current study was the differential response to atorvastatin in total cholesterol and non-HDL cholesterol. The Gly247Gly genotype had been previously shown to be protective against statin intolerance and myalgia (Siddiqui et al., 2017) compared to the Asp247Asp genotype. In our study the Gly247Gly genotype group similarly showed no significant post-statin increase in CK, whereas the Asp247Asp group did. Interestingly, participants with the Gly247Gly genotype also had better total and non-HDL cholesterol-lowering effect than the group with Asp247Asp genotype. There was also a significant interaction between treatment and genotype. The results were replicated in observational cohort of patients treated with statin therapy from the Tayside bioresources.

The two main limitations of this study were the limited sample size and the short study duration. Even though there was a trend of differential response in the average number of CD4+/Foxp3+ T cells between the genotype groups following treatment with statin compared to treatment with placebo, this effect was not significant, likely/possibly due to limited sample size. Further, the study participants were healthy and not suffering from cardiovascular diseases, which may influence the level of low-grade inflammation and modulate the immunological response to statin therapy as to attenuate genetic effects. While this might limit the generalizability of our findings, the age of the study subjects represents the typical age of patients prescribed statins (Melhem et al., 2021). Statin ADRs, and statin-induced myopathy among them, are more common in aged individuals and senescent animal models (Camerino et al., 2016; Arrigoni et al., 2017; Ward et al., 2019; Belch et al., 2021). The potential mechanism for the effect of *LILRB5* on statin tolerance is based on the reduced expression of Foxp3+ T regulatory cells in Asp247Asp carriers, resulting in poorer muscle repair. The reduced expression of T-regulatory cells is also a known effect of aging (Ward et al., 2019). It is therefore possible that in an aging body the dual impact of reduced T regulatory cells and the interaction between this genotype and statin use has an even more profound effect. Given the duration of treatment in this trial, we are not able to make any assessment of the long-term effect of atorvastatin by genotype. The lack of a mechanistic pathway for the *LILRB5* variant to show this effect is a limitation.

One of the main strengths of the current study is that it was performed in a prospective, placebo-controlled double-blind setting. The subjects and the investigators were blinded to both the genotype and the treatment phase during the trial. Moreover, we used a cross-over design, thus allowing to compare both subjective and objective measurements of atorvastatin effects within the same subjects compared to placebo. In order to limit interindividual variability, we recruited only white subjects of European ancestry who were healthy and not on any concomitant medications. The dose used in the current study was 80 mg atorvastatin, which is clinically relevant and represents the current guidelines in the UK for maximum therapeutic dose (National Institute for Health and Care Excellence, 2016). Moreover, the duration of 4 weeks of statin administration was long enough to reach steady state and to achieve significant changes in lipid levels. Thus, the current study can be considered to represent the clinical use of statin therapy initiation.

Functional characterization of the Gly247Asp genotype is limited. Previous evidence showed a dominant effect of the variant on CK levels [Asp247Asp (T/T) vs. Gly247Asp (T/C) + Gly247Gly (C/C)]. Gly247Asp is a common variant with a mean allele frequency of about 40% in White Europeans; for a person to be homozygous for this allele, the likelihood is 0.16, i.e., 16% of the population should have the Gly247Gly (C/C) genotype. On the other hand, 36% should have the Asp247Asp (T/T) genotype and 48% should have the Gly247Gly (T/C) genotype. We recruited only those homozygous for Gly247Asp [Asp247Asp (T/T) or Gly247Gly (C/C)], excluding heterozygous carriers, to increase the probability of detecting the effect of *LILRB5* on statin tolerance and changes in T regulatory cells aggregation. Further studies are required to characterize the functional implications of this genetic variant.

Our observed effect of *LILRB5* genotype on non-HDL cholesterol response is supported by previous findings from the Tayside bioresource. In the study by Melhem et al. non-HDL -cholesterol response was defined as lowest measured non-HDL cholesterol, after a minimum of 4 weeks of statin therapy up to a maximum of 6 months after commencement of statin therapy (Melhem et al., 2021). Individuals with the C/C genotype had an absolute reduction of 0.05 mmol/L (0.01,0.08, *p* < 0.05) greater than carriers of the T/T genotype. In a previous study, the *LILRB5* Asp247Asp genotype was associated with increased risk of statin intolerance defined based on prescription patterns and raised CK levels or prescription patterns only (Siddiqui et al., 2017). Summary statistics from the UK Biobank show an effect of lower total, LDL and HDL-cholesterol for C variant carriers (Elsworth et al., 2020). However, these estimates are unadjusted for medication use. Our results are robust to the possibility of lower cholesterol at baseline as we have performed paired tests to control for intra-individual variability.

The role of this variant and indeed other immunological variants has not been explored in the context of pharmacokinetics and pharmacodynamics of statins. A combination of large observational studies and clinical trials would be required to confirm our findings. Further research is needed to establish if the effect of the *LILRB5* genotype is driven by non-adherence to statin therapy or by a more direct effect on lipid response.

## Data Availability

The original contributions presented in the study are included in the article/Supplementary Material, further inquiries can be directed to the corresponding author.
