# Supplementary material for: The cholesterol-lowering effect of statins is modified by LILRB5 intolerance genotype: Results from a recruit-by-genotype clinical trial
Source: Front Pharmacol. 2023 Mar 14;14:1090010. doi: 10.3389/fphar.2023.1090010 (PMC10043296; doi:10.3389/fphar.2023.1090010)
Supplement: Supplementary file 1 [file Table1.DOCX]

# Supplementary Materials and Methods

## Methods

### 1. Consort diagram


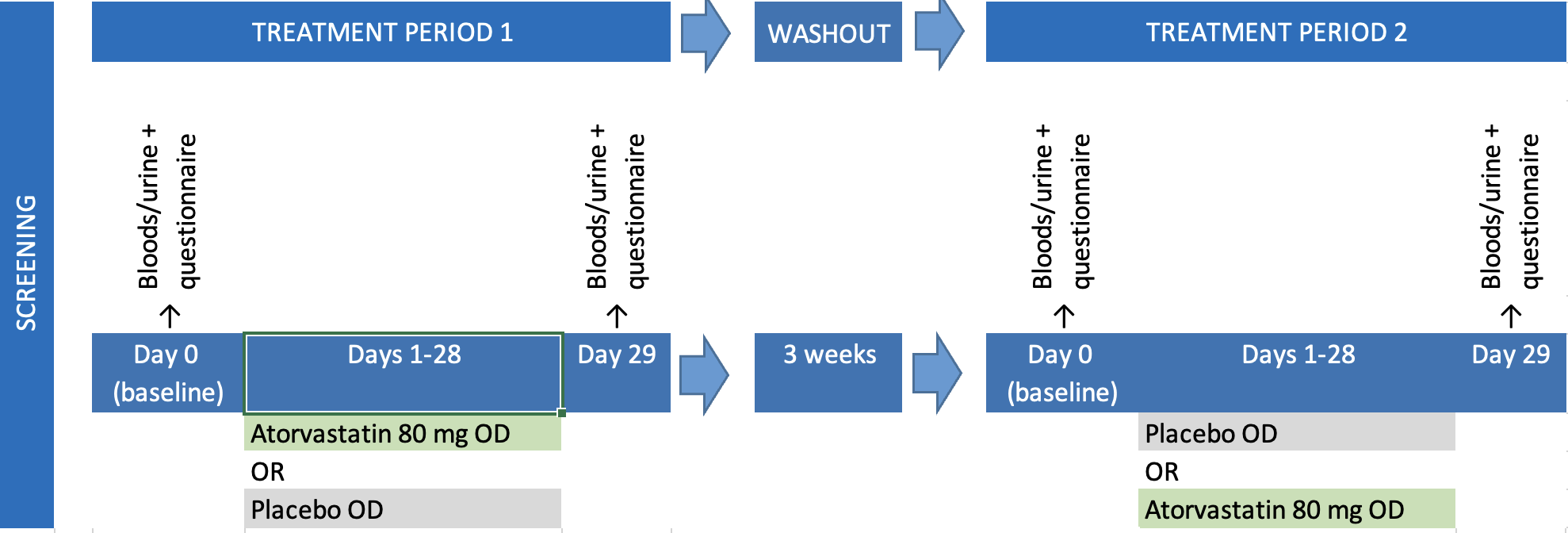


Supplementary Figure 1. Immunostat study design

## Results

## Creatinine phosphokinase response to Atorvastatin therapy by *LILRB5* Asp247Gly genotype

Supplementary Table 1 Differences in creatinine kinase (CK) response to therapy by genotype

| **Genotype** | **Treatment** | **Median (IQR)** | **N** |
| --- | --- | --- | --- |
| **Genotype C/C** | Placebo | -1 (67) | 7 |
|  | Atorvastatin | 12.5 (26) | 7 |
| **Genotype T/T** | Placebo | -6 (127.5) | 8 |
|  | Atorvastatin | 14.5 (37) | 8 |

## Total cholesterol response to atorvastatin therapy by *LILRB5* Asp247Gly genotype

Supplementary Table 2. Differences in total cholesterol response to therapy by genotype

| **Genotype** | **Treatment** | **Mean** | **Std Deviation** | **N** |
| --- | --- | --- | --- | --- |
| **Genotype C/C** | Placebo | 0.269 | 0.598 | 7 |
|  | Atorvastatin | -2.434 | 0.652 | 7 |
| **Genotype T/T** | Placebo | -0.056 | 0.303 | 8 |
|  | Atorvastatin | -1.304 | 0.815 | 8 |

Supplementary Table 3. Statistical association between total cholesterol response, genotype, and treatment

| **Source** | ***df*** | **MS** | ***F*** | ***p*** | **Effect Size** |
| --- | --- | --- | --- | --- | --- |
| **Treatment** | 1 | 29.12 | 73.16 | <0.0001**** | .67 |
| **Genotype** | 1 | 1.212 | 3.272 | 0.0936 | .028 |
| **Treatment x Genotype** | 1 | 3.954 | 9.930 | 0.0077** | .092 |

## Non-HDL cholesterol response to atorvastatin therapy by *LILRB5* Asp247Gly genotype

Supplementary Table 4. Differences in non-HDL cholesterol response to therapy by genotype

| **Genotype** | **Treatment** | **Mean** | **Std Deviation** | **N** |
| --- | --- | --- | --- | --- |
| **Genotype C/C** | Placebo | 0.194 | 0.539 | 7 |
|  | Atorvastatin | -2.434 | 0.624 | 7 |
| **Genotype T/T** | Placebo | -0.024 | 0.381 | 8 |
|  | Atorvastatin | -1.300 | 0.760 | 8 |

Supplementary Table 5. Statistical association between non-HDL cholesterol, genotype, and treatment

| Source | df | MS | F | p | Effect Size |
| --- | --- | --- | --- | --- | --- |
| **Treatment** | 1 | 25.74 | 65.14 | <0.0001**** | .69 |
| **Genotype** | 1 | 0.981 | 3.187 | 0.0976 | .026 |
| **Treatment x Genotype** | 1 | 2.516 | 6.366 | 0.0255* | .067 |
